# Is it possible to predict the length of time on continuous hemodialysis?

**DOI:** 10.5935/0103-507X.20220029-en

**Published:** 2022

**Authors:** Lucas Gobetti da Luz, Maurício Lutzky, Daniel Sganzerla, Giselle Calovi Pratini, Cassiano Teixeira

**Affiliations:** 1 Service of Nephrology, Hospital Moinhos de Vento - Porto Alegre (RS), Brazil.; 2 Project Office, Hospital Moinhos de Vento - Porto Alegre (RS), Brazil.; 3 Intensive Care Unit, Hospital Moinhos de Vento - Porto Alegre (RS), Brazil.

**TO THE EDITOR,**

Acute kidney injury is a common organ dysfunction observed in intensive care units (ICUs), with a prevalence ranging from 2.5% to 92.2%, according to the population studied.^(1,2)^ It is estimated that 0.8% to 59.0% of the patients admitted to an ICU require renal replacement therapy (RRT) at some point during hospitalization.^(1)^ Nevertheless, these patients have worse short- and long-term outcomes.^(3)^ The main modalities of RRT used in the ICU are intermittent hemodialysis, extended hemodialysis, and continuous therapy (which can be continuous hemodialysis, continuous hemofiltration, or continuous hemodiafiltration).^(4)^ Neurocritical issues and dysnatremias are the main formal indications for continuous therapies; however, from a practical perspective, centers that have continuous therapies available also use them in patients in need of vasoactive drugs since the fluid removal rate per hour is lower than that in intermittent therapies, generating less hemodynamic instability.^(5)^

The coronavirus 2019 (COVID-19) pandemic caused approximately 22% of critically ill patients to need RRT, resulting in a greater demand for beds capable of offering RRT in ICUs.^(6)^ Due to operational and clinical facilities, the National Institute of Health (NIH) recommended the preferential use of continuous therapies over intermittent therapies in these patients.^(7)^ This further increased the demand for trained professionals and supplies. Given this scenario, tools that can predict the time a patient spends in RRT have become necessary, either for clinical purposes or supply and therapy planning purposes.

Thus, since the supplies used for these therapies are different from each other, as well as the needs related to logistical planning for their acquisition, an adequate *timing* prediction for the transition between the therapies (continuous to intermittent) could enable improved management of therapy resource allocations in ICUs. Thus, the present study included 480 patients diagnosed with septic shock (norepinephrine-dependent ≥ 0.5µg/kg/minute) who required continuous RRT (Kidney Disease: Improving Global Outcomes - KDIGO ≥ 2). Data were collected retrospectively from a single ICU, and the time in continuous therapy was evaluated according to the number of organ dysfunctions present in the first 72 hours of admission. The organ dysfunctions were classified as follows: gastrointestinal (need for total parenteral nutrition), neurological (diagnosis of *delirium* evaluated by means of Confusion Assessment Method for the Intensive Care Unit - CAM-ICU), respiratory (need for invasive or noninvasive ventilatory support), and hematological (need for transfusion of blood products). The ICU in the study already used continuous RRT as the first choice for patients with classical indications and for those who require moderate doses of vasoactive drugs during dialysis sessions. Therefore, there was no limitation due to a lack of expertise or resources that could impact the prescription of therapy.

In the study, the quantitative variables were described as medians and interquartile ranges. The comparison between the time on continuous hemodialysis, in days, and the presence or absence of each organ dysfunction was performed using the Mann-Whitney U test. The comparison between the number of days on continuous hemodialysis and the number of organ dysfunctions was performed with the Kruskal-Wallis test, treating the number of days categorically, and the relationship between the variables was also verified using the Spearman’s correlation.

Individually, all organ dysfunctions were related to a longer duration on continuous dialysis therapy (Table 1). Two organ dysfunctions were present in 6.3% of the patients, three dysfunctions were present in 24.6%, four dysfunctions were present in 36.7%, five dysfunctions were present in 28.5%, and six dysfunctions were present in 4.0%.

**Table 1:** Classification and incidence of acute organ dysfunction

Type of organ dysfunction	Occurrence rate (%)	Time on continuous hemodialysis (in days)		
		With organ dysfunction	No organ dysfunction	p value
Gastrointestinal	29/480 (6.0)	17 (11 - 28)	6 (3 - 12)	< 0.001
Neurological	330/480 (68.8)	9 (5 - 15)	2 (1 - 3)	< 0.001
Respiratory	444/480 (92.5)	7 (3 - 13)	1 (1 - 2)	< 0.001
Hematological	154/480 (47.2)	15 (11 - 20)	4 (2 - 7)	< 0.001

The results are expressed as total n/n (%) or median and interquartile range.

**Figure 1:**
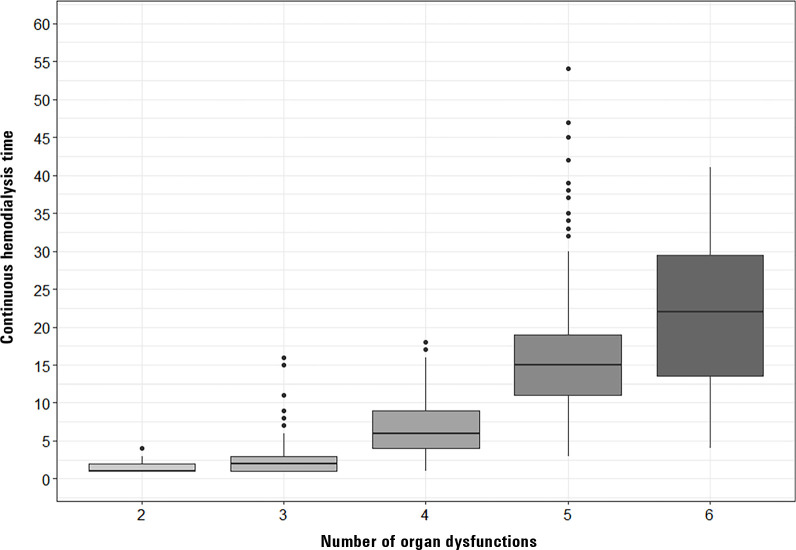
Days of continuous renal replacement therapy in relation to the number of acute organ dysfunctions developed in the first 72 hours of admission to the intensive care unit. Spearman correlation: 0.81 (0.77 - 0.84).

Figure 1 shows the correlation between the number of organ dysfunctions and the median duration of continuous renal replacement therapy. The number of organ dysfunctions was associated with time on continuous hemodialysis, with a Spearman correlation of 0.81 (0.77 - 0.84).

Thus, from the data obtained, it is believed that the number of organ dysfunctions present within the first 72 hours of admission of patients with septic shock can predict the duration of continuous RRT. Although incipient, this could be a useful and practical tool for the organization of an ICU and its dialysis resources. Although retrospective, the objective of this study was to provide a foundation for assisting resource management based on clinical criteria focused on nephrointensivism.
